# Global rotavirus vaccine introductions and coverage: 2006 – 2016

**DOI:** 10.1080/21645515.2018.1470725

**Published:** 2018-06-18

**Authors:** Alice J. Abou-Nader, Molly A. Sauer, A. Duncan Steele, Jacqueline E. Tate, Deborah Atherly, Umesh D. Parashar, Mathuram Santosham, E. Anthony S. Nelson

**Affiliations:** a Department of Paediatrics, The Chinese University of Hong Kong, Hong Kong S.A.R.; b Department of International Health, Johns Hopkins University, Baltimore, Maryland, United States; c Bill and Melinda Gates Foundation, Enteric and Diarrheal Diseases, Seattle, Washington, United States; d Centers for Disease Control and Prevention, Division of Viral Diseases, Atlanta, Georgia, United States; e PATH, Center for Vaccine Innovation and Access, Seattle, Washington, United States

**Keywords:** developed countries, developing countries, Gavi-eligible countries, national immunization program, new vaccine introduction, Rotavirus vaccine

## Abstract

An estimated 215,000 children died of rotavirus infections in 2013, accounting for 37% of diarrhea-related deaths worldwide, 92% of which occurred in low and lower-middle income countries. Since 2009 the World Health Organization (WHO) recommends the use of rotavirus vaccines in all national immunization programs. This review compares rotavirus vaccine (RV) introductions and vaccine coverage by region, country income status and Gavi-eligibility from 2006–2016. Gross National Income data from the World Bank and surviving infant population from United Nations Population Division was obtained for 2016. Data from WHO were collected on rotavirus vaccine coverage, national immunization schedules, and new vaccine introductions for 2016 while estimated rotavirus deaths were collected for 2013, the last year of available WHO data. As of December 2016, the majority of countries (57%, 110/194) had not introduced universal rotavirus vaccine despite WHO's 2009 recommendation to do so. Countries in the WHO African region had the greatest proportion of introductions (37%, 31/84) by December 2016 and a great majority of these (77%, 24/31) were supported by new vaccine introduction (NVI) grants from Gavi. Almost half (48%) of global introductions were in low and lower-middle income Gavi-eligible and Gavi-graduating countries. Conversely, countries in the Southeast Asia WHO region and those not eligible for Gavi NVI support have been slow to introduce rotavirus vaccine. High-income countries, on average, had poorer rotavirus vaccine coverage compared to low and lower-middle income countries. The over-representation of African countries within the Gavi subset and high estimated rotavirus deaths in these African countries, likely explains why introduction efforts have been focused in this region. While much progress has been made with the integration and implementation of rotavirus vaccine into national immunization programs, 110 countries representing 69% of the global birth cohort had yet to introduce the vaccine by December 2016.

## Introduction

Deaths caused by rotavirus infection are largely preventable through immunization. An estimated 215,000 children died of rotavirus infections in 2013, accounting for 37% of diarrhea-related deaths worldwide.^1^ Approximately half (49%) of these deaths were in India, Nigeria, Pakistan, and the Democratic Republic of Congo,^1^ which are low and lower-middle income countries (LICs and LMICs) eligible to receive financial assistance from Gavi through new vaccine introduction (NVI) grants. This review summarizes global rotavirus vaccine introductions during the period 2006 to 2016 according to country income group, World Health Organization (WHO) region, and Gavi-eligibility status.

There are two WHO pre-qualified rotavirus vaccines^2^ that were licensed internationally in 2006.^3^ Merck's pentavalent reassortant human bovine vaccine (RotaTeq) and GlaxoSmithKline's monovalent vaccine (Rotarix) were widely licensed following large clinical trials in the Americas and Europe, leading to a limited recommendation by WHO in 2006 for regional introduction.^3^ In 2009, following efficacy studies in LICs and LMICs in Africa and Asia, WHO recommended that all countries introduce rotavirus vaccine into their National Immunization Programs (NIP).^4^ WHO position papers also recommend that National Immunization Technical Advisory Groups (NITAGs) consider cost-effectiveness and the local disease burden when considering whether rotavirus vaccine should be introduced into the NIP. Although rotavirus vaccines have higher vaccine efficacy in developed, high-income countries (HICs) than in LICs and LMICs, the public health impact of the vaccine is often greater in low-resource settings.^5^ In 2013, WHO updated its rotavirus vaccine position paper and removed the age restrictions for vaccination.^6^ As a result, the rotavirus vaccine schedule follows that of the Diphtheria Tetanus Pertussis (DTP) vaccine, irrespective of whether countries select the two-dose Rotarix or three-dose RotaTeq vaccine. This review aims to better understand variations in rotavirus vaccine introductions and coverage by WHO region, country income status, and Gavi-eligibility and to assess the integration of universal rotavirus vaccine introduction into NIPs.

## Methods and data sources

### Gavi-eligible, transitioning, and graduating countries

Gavi's New Vaccine Introduction (NVI) grants have supported eligible countries to introduce rotavirus vaccine. These grants are contingent on a country's Gross National Income (GNI) per capita with eligibility defined as an average three-year GNI less than or equal to US$ 1,580 per capita.^7^ As GNI increases, a country moves through Gavi's different eligibility phases until reaching the transition phase, when GNI exceeds the eligibility threshold. In this analysis 73 Gavi-eligible countries in Phase III (2010-2015) were divided into two sub-groups according to published information for Phase IV (2016–2020): 52 Gavi-eligible and 21 Gavi-transitioning or Gavi-graduating countries (Appendix 1).^8^^,^^9^ Nicaragua and Papua New Guinea were listed in Phase IV in both eligible and transitioning groups but were classified as transitioning countries in this analysis.

### World bank and united nations databases

The World Bank Indicator Database and United Nations Population database provide demographic information for the 194 United Nations member states. The following information was obtained and summarized for 2016:

2.1) The World Bank's 2016 GNI per Capita (Atlas Method, current US$) was used to classify countries into the following income groups: low income countries (LICs) with GNI per capita ≤ US$1,005; lower-middle income countries (LMICs) with GNI per capita US$1,006 to ≤ US$3,955; upper-middle income countries (UMICs) with GNI per capita US$3,956 to ≤ US$12,235; and high income countries (HICs) with GNI per capita > US$12,235.^10^^,^^11^ Data from previous years were imputed for 20 countries (10%, 20/194) that had missing GNI data for 2016. Four countries (Cook Islands, DPR Korea, Niue, and Somalia) had no historical GNI data available for imputation for 2016. To include these countries in the analysis, total Gross Domestic Product for 2015 were collected from the United Nations database and divided by the total population to assign an income group to these countries.^12^

2.2) Total population, infant mortality, and birth rates were obtained from the United Nations Population database to calculate the surviving infant population for 2016 using the following formula^13^:

[Total Population × (Birth Rate/1,000)] – [(Total Population × (Birth Rate/1,000)) × (Infant Mortality/1,000)]

Medium variant crude birth rates and infant mortality estimates for 2015–20 were collected for the 194 United Nations member states. Population data from the Central Intelligence Agency World Factbook were obtained for the 11 countries (Andorra, Cook Islands, Dominica, Marshall Islands, Monaco, Nauru, Niue, Palau, San Marino, Saint Kitts and Nevis, Tuvalu) with missing data from United Nations sources.^14^

### WHO vaccine introduction and coverage datasets

Whether rotavirus vaccine was included in NIP, year of rotavirus vaccine introduction, rotavirus and DTP vaccine coverage (for the first dose and last dose of the series) and estimated rotavirus deaths for 2013 were extracted from the following WHO datasets.

#### WHO/UNICEF – joint reporting form (JRF)

Since 1998, Ministries of Health of United Nations member states annually complete the JRF, a template with indicators related to the country's maternal and child health. This analysis compiles data from three different datasets that were created from the JRF^15^: 1) Year of New Vaccine Introduction; 2) National Immunization Schedules and; Vaccine Coverage Estimates. These three JRF datasets were compared and used to identify the subset of countries that had universally introduced rotavirus vaccine by December 2016 (Appendix 1, Appendix 2).

3.1) New Vaccination Introduction dataset: The year of rotavirus vaccine introductions was downloaded from hyperlink item “6.2 year of vaccine introduction” on WHO/UNICEF JRF data, statistics, and graphics website.^16^ This data set was used to define ‘introducing’ and ‘non-introducing’ countries. Countries that reported introducing rotavirus vaccine in 2016 or before were considered ‘rotavirus vaccine introducers’ while the remaining were listed as ‘non-introducers’ (Table 1, Table 2). Canada (2010), India (2016), Italy (2016), Philippines (2012), Pakistan (2016) and Sweden (2014) are listed as having partially introduced the vaccine into their countries but had not offered the vaccine nation-wide. Seven countries (Belize, Central African Republic, Cote d'Ivoire, Lesotho, Pakistan, Seychelles, Uganda) reported scheduled rotavirus vaccine introductions for 2017, and Nigeria for 2018.

**Table 1.** Countries with universal rotavirus vaccine introduction on or before 2016.

WHO Regions	2006	2007	2008	2009	2010	2011	2012	2013	2014	2015	2016	Total
Africa	—	—	—	South Africa	—	—	Botswana Ghana Malawi Rwanda	Burkina Faso Burundi Gambia Tanzania Zambia	Angola, Cameroon, Congo, Eritrea, Ethiopia, Kenya, Madagascar, Mauritania, Namibia, Niger, Senegal, Sierra Leone, Togo, Zimbabwe	Guinea Bissau Mali Mauritius Mozambique Swaziland	Liberia São Tomé and Príncipe	31
Americas	Brazil El Salvador Nicaragua Panama United States Venezuela	Ecuador Mexico	Bolivia	Colombia Honduras Peru	Guatemala Guyana Paraguay	—	Dominican Republic	—	Haiti	Argentina	—	18
East Mediterranean	—	—	Bahrain	Qatar	Morocco	Sudan	Iraq Yemen	Libya Saudi Arabia	Djibouti United Arab Emirates	Jordan	—	11
Europe	Austria Luxembourg	Belgium	—	Finland	Israel	Greece	Armenia Moldova	Georgia Germany United Kingdom	Estonia Norway Uzbekistan	Latvia Tajikistan	Ireland	17
Southeast Asia	—	—	—	—	—	—	—	—	—	—	—	0
Western Pacific	—	Australia	Micronesia Palau	Marshal Islands	—	—	Fiji	—	New Zealand	Kiribati	—	7
2-dose schedule	6	1	2	5	2	1	9	6	19	7	1	59
3-dose schedule	2	2	2	2	3	—	1	3	2	3	2	22
Doses depends on presentation	—	1	—	—	—	1	—	1	—	—	—	3
Total	8	4	4	7	5	2	10	10	21	10	3	84

a Rotavirus vaccine introduction year gathered from the WHO/UNICEF – Joint Reporting Form (JRF) New Vaccine Introduction dataset.

b Two-dose rotavirus vaccine schedule reported in JRF (presumed to be monovalent rotavirus vaccine Rotarix produced by GlaxoSmithKline).

c Three-dose rotavirus vaccine schedule reported in JRF (presumed to be pentavalent rotavirus vaccine RotaTeq produced by Merck).

d Countries that reported a three-dose schedule in the JRF but specified that the third dose depends on the presentation of the vaccine.

**Table 2.** WHO region-specific summary of rotavirus vaccine introductions by country income-group, 2016.

	Income Groups for 2016 as Defined by the World Bank										GAVI Phase IV			
	LICs		LMICs		UMICs		HICs		Total		Eligible		Transitioning	
WHO Regions	(n/N)	%	(n/N)	%	(n/N)	%	(n/N)	%	(n/N)	%	(n/N)	%	(n/N)	%
Africa	18/26	69%	9/13	69%	4/7	57%	0/1	0%	31/47	66%	24/35	69%	2/2	100%
Americas	1/1	100%	5/5	100%	11/20	55%	1/9	11%	18/35	51%	1/1	100%	4/5	80%
East Mediterranean	0/2	0%	5/9	56%	2/4	50%	4/6	67%	11/21	52%	3/6	50%	0/0	0%
Europe	0/0	0%	5/7	71%	0/14	0%	12/32	38%	17/53	32%	1/2	50%	4/6	67%
Southeast Asia	0/2	0%	0/7	0%	0/2	0%	0/0	0%	0/11	0%	0/5	0%	0/4	0%
Western Pacific	0/0	0%	2/10	20%	2/9	22%	3/8	38%	7/27	26%	0/3	0%	1/4	25%
Total	19/31	61%	26/51	51%	19/56	34%	20/56	36%	84/194	43%	29/52	56%	11/21	52%

a Number of countries that introduced the rotavirus vaccine into the National Immunization Program on or before December 2016, WHO/UNICEF – Joint Reporting Form, New Vaccine Introduction dataset.

b Lower Income Countries (LICs) defined as GNI per capita ≤ US$1,005; Lower-middle Income Countries (LMICs) defined as US$1,006 ≤ GNI per capita ≤ US$3,955; Upper-middle Income Countries (UMICs) defined as US$3,956 ≤ GNI per capita ≤ US$12,235; Higher Income Countries (HICs) defined as GNI per capita > US$12,235.

c List of Phase IV countries that receive Gavi financial support for new vaccine introductions from 2016–2020.

d Includes the five (Bhutan, Honduras, Mongolia, Sri Lanka, Ukraine) Phase III (2011-15) Gavi-graduating countries.

3.2) National Vaccination Schedule dataset: The Excel-based dataset was downloaded from the “6.1 National Immunization Schedule” hyperlink on the WHO/UNICEF JRF data, statistics, and graphics website.^16^ Data were obtained for the 100 countries that had reported including rotavirus vaccine in their NIP schedule in 2016. Eight countries (Canada, India, Italy, Mali, Romania, Russia, Sweden, Thailand) offered the vaccine to only part of their population, either through the private sector, in a specific geographic area, or depending on the country's disease burden. Mali also reported universally introducing the vaccine in 2015 in the New Vaccination Introduction dataset and was therefore considered an introducing country.

Vaccine schedules for DTP vaccine were compared with the rotavirus vaccine schedules: 1) two-dose schedule countries (presumed Rotarix) were matched with the DTP schedule for the first two doses; and 2) three-dose schedule countries (presumed RotaTeq) were matched with the three-dose DTP schedule. It is possible that countries might have introduced both types of rotavirus vaccine but only those reported to the JRF as part of the national immunization schedule were considered. Belgium, Germany and Greece reported three-dose schedules but stated the third dose was determined by vaccine presentation (indicating both Rotarix and RotaTeq introduced).

3.3) Vaccination coverage datasets: Listed as hyperlink items “4.1 Official Country Reported Coverage Estimates Time Series” and “4.5 WHO/UNICEF Estimates on National Immunization Coverage (WUENIC)”, these Excel-based datasets on country-reported and WUENIC vaccine coverage estimates were downloaded from the WHO/UNICEF – JRF data, statistics, and graphics website.^16^ Country-reported estimates of rotavirus vaccine coverage for the first (Rota1) and last dose in the series (second or third dose, Rota2-3) were abstracted for 2016. From 2008–2016, 89 countries reported rotavirus vaccine coverage at least once.

*WHO/UNICEF Estimates of National Immunization Coverage (WUENIC) and country-reported estimates of Rota2-3 coverage*: WUENIC estimates are available for the first and third dose of the (DTP) vaccine but only for last dose of rotavirus vaccine. Country-reported and WUENIC estimates for rotavirus vaccine coverage for introducing countries were compared. A great majority (71%, 50/70) of reporting countries had the same rotavirus vaccine coverage estimates, 7 (Bolivia, Botswana, Brazil, Congo, Djibouti, Fiji, Micronesia) had WUENIC estimates that were on average 15% above country-reported coverage estimates, and 13 (Angola, Estonia, Ethiopia, Haiti, Honduras, Madagascar, Mali, Mauritania, Nicaragua, Niger, São Tomé and Príncipe, Yemen, Zambia) had WUENIC coverage estimates that were on average 28% lower than country-reported estimates. Country-reported estimates of rotavirus vaccine were selected over WUENIC data for our main analysis since they had coverage data reported for both the first and last dose of the rotavirus vaccine.

*WUENIC-reported and country-reported DTP coverage estimates*: 48 countries had country-reported coverages of DTP1 and DTP3 that were the same as WUENIC estimates, 7 had country-reported coverages that were 9% (DTP1) and 8% (DTP3) lower than WUENIC estimates, and 15 countries had country-reported coverage that were 9% (DTP1) and 16% (DTP3) higher than WUENIC estimates. Country-reported estimates were also selected for our main analysis.

*Coverage and drop out as utilization and access performance indicators*: Vaccine coverage provides insight on the performance of NIPs. WHO's Reaching Every District Approach outlines a set of NIP performance indicators using DTP1 coverage and the drop-out rate between DTP1 and DTP3 (percent difference between the first and third dose of the DTP vaccine, formula (DTP1-DTP3/DTP1) × 100 to evaluate the utilization and access to the vaccine (Appendix 3).^17^ A well-functioning NIP with adequate utilization of and access to vaccination was defined as having a low drop-out rate (below 10%) and a high DTP1 vaccine coverage (above 80%). In contrast, poor utilization and access are reflected by a high drop-out rate (above 10%) and low DTP1 coverage (below 80%). These indicators are used to assess performance of vaccination services in general, rather than individual vaccines. However, since rotavirus vaccination follows the same immunization schedule as the DTP vaccine, we explored in this review whether these indicators could be useful in quantifying the utilization of and access to the rotavirus vaccine in introducing countries. We used Rota1 coverage to represent the target population's access to this vaccine. Utilization of rotavirus vaccine was calculated as the drop-out rate between the first and last dose of the rotavirus vaccine course. Four indicators were generated based on these Rota1 coverage and drop-out (Appendix 3). Coverage values for the first and last dose of the rotavirus (Rota1 and Rota2-3) and diphtheria, pertussis, tetanus (DTP1 and DTP3) vaccines were also matched to calculate the percent difference between the two vaccines. This was calculated by dividing the difference between DTP and rotavirus vaccine coverage by the DTP coverage for each respective dose. Coverage of the second dose of rotavirus vaccines following a two-dose schedule was matched with DTP3 vaccine coverage. Only countries with complete reporting on DTP and rotavirus vaccine coverages (N = 70) for 2016 were included in this analysis.

4) *Rotavirus mortality*: The estimated number of rotavirus deaths for 2013 were obtained from the WHO Immunization, Vaccines and Biologicals website.^18^ Data from 2000–2013 for children less than 5 years of age were made available through an Excel-based dataset downloaded from the website's resource section.

### Data analysis

Statistical Software Stata14/IC was used for analysis. Summary statistics were calculated for each variable, disaggregating between the subset of rotavirus vaccine introducing countries, non-introducing countries, WHO region, income-group, and Gavi-eligibility. The surviving infant population was divided into three groups: immunized, unimmunized or untargeted. Infants residing in rotavirus vaccine introducing countries were either immunized or unimmunized while infants residing in non-introducing countries were labeled as untargeted for not having access to the vaccine by December 2016. Immunized infants were assumed to have received at least one dose of vaccine and this number was calculated by multiplying the surviving infant population by the reported Rota1 coverage. The unimmunized infants were assumed to have never received rotavirus vaccination and this number was calculated by subtracting the number of immunized infants by total surviving infant population for each country and region.

## Results

### Rotavirus vaccine introduction, 2006–2016

Rotavirus vaccine introductions varied by country income group and WHO region during the period 2006–2016. 84 countries (43%, 84/194) had reported introducing rotavirus vaccines (Rotarix 59, and RotaTeq 25) into their NIPs by December 2016 (Table 1). In all, 81 countries had complete data for all three JRF datasets with Ireland, Mozambique, and Norway having missing data for either rotavirus vaccine coverage or inclusion of the vaccine in their NIP (Appendix 2). The majority (79%, 23/29) of Gavi-eligible introducing countries reported using Rotarix, whereas RotaTeq was predominantly used in HICs (60%, 12/20) (Table 1). A large number of Rotarix introductions (25%, 21/84) took place in 2014 (Table 2) and 67% (14/21) were in the WHO African region.

The African WHO region had the greatest proportion of introductions (37%, 31/84) with the great majority (84%, 26/31) of these countries doing so with NVI-grants from Gavi. This region also had the highest overall percent (66%, 31/47) of introductions while the Southeast Asia WHO region had the lowest (0%, 0/11) (Table 2). The Eastern Mediterranean region had the second highest percentage (52%, 11/21) of vaccine introductions, followed by the Americas region (51%, 18/35). In the Americas, 33% (6/18) of introductions took place in 2006, the first year of vaccine licensure (Table 2). A great majority (71%, 12/17) of the non-introducers in the Americas were small Caribbean islands (Antigua and Barbuda, Bahamas, Barbados, Belize, Cuba, Dominica, Grenada, Jamaica, Saint Kitts and Nevis, Saint Lucia, Saint Vincent and the Grenadines, Trinidad and Tobago). Furthermore, the majority (61%, 11/18) of these rotavirus vaccine introductions took place in UMICs of which only Guyana was eligible to receive Gavi NVI grants. All countries in the Americas, except for the United States and Canada, have access to negotiated fixed vaccine pricing through the region's pooled procurement mechanism, the Revolving Fund. In contrast, none of the 14 UMICs in the European region introduced rotavirus vaccine, potentially because of the vaccine's higher price as countries independently negotiate pricing with pharmaceutical companies. Most of Europe's (71%, 12/17) rotavirus vaccine introductions took place in non-Gavi HICs (Table 2). Rotavirus vaccine introductions in Europe's 5 LMICs were with Gavi's financial assistance. Kyrgyzstan was the only Gavi-eligible LMIC in the region to not have introduced the vaccine.

Thus globally, introductions occurred in 61% (19/31) of LICs, 51% (26/51) of LMICs, 34% (19/56) of UMICs, and in 36% (20/56) of HICs (Table 2). Most of the introductions in non-Gavi countries were in the Americas (31%, 14/45) and Europe (27%, 12/45). Overall 48% (40/84) of global rotavirus vaccine introductions took place in Gavi-eligible and Gavi-transitioning countries, accounting for more than half (55%, 40/73) of the eligible countries receiving some form of financial assistance from Gavi. Of the five Phase III Gavi-transitioning countries (Bhutan, Honduras, Mongolia, Sri Lanka, and Ukraine), only Honduras had introduced rotavirus vaccine by December 2016.

### Rotavirus vaccine coverage and performance, 2016

*Country-reported rotavirus vaccine and DTP vaccine coverage*: Out of the 84 introducing countries, 77 reported coverage data for the first dose and 80 for the last dose of rotavirus vaccine. In all, 76 countries had complete data for both doses, out of which 9% of countries (7/76; Australia, El Salvador, Iraq, Israel, Moldova, South Africa, United Kingdom) had data that was inconsistent or incomplete (i.e. Rota1 coverage was less than Rota2-3 coverage or Rota1 coverage was missing while Rota2-3 data was reported, and vice-versa). Data for these flagged observations were imputed with the averaged rotavirus vaccine coverage for that country's respective income-region. The same imputation procedure was conducted for the three countries (4%, 3/76; Austria, Ireland, Norway) that did not report rotavirus vaccine coverage for either dose. Country-reported DTP vaccine coverages were inconsistent and/or incomplete for 6 countries (Argentina, Ecuador, El Salvador, Iraq, Palau, Paraguay). Imputed rotavirus and/or DTP coverage data for the 14 countries with inconsistent or missing data were only used to calculate the number and percent of infants immunized, unimmunized and untargeted. These countries were excluded from the vaccine coverage summaries of the 70 rotavirus introducing countries that had consistent and complete reporting for the first and last dose of the rotavirus and DTP vaccine series (Table 3).

**Table 3.** Income-specific summary of country-reported rotavirus and diphtheria, pertussis, tetanus (DTP) vaccine coverage estimates, 2016.

	Income Groups for 2016 as Defined by the World Bank										GAVI Phase IV			
Variables	LICs (n = 19)		LMICs (n = 24)		UMICs (n = 14)		HICs (n = 13)		Total (N = 70)		Eligible (n = 29)		Transitioning (n = 10)	
Average Country-Reported Rotavirus Vaccine Coverage														
Dose 1	91%		89%		89%		84%		89%		91%		90%	
Dose 2–3	85%		83%		80%		80%		82%		84%		86%	
Drop-out	8%		8%		11%		6%		8%		8%		4%	
Average Country-Reported DTP Vaccine Coverage														
Dose 1	95%		94%		96%		98%		95%		94%		94%	
Dose 3	89%		88%		89%		97%		90%		89%		90%	
Drop-out	6%		6%		7%		1%		5%		6%		4%	
Percent Difference between DTP & Rota Vaccine Coverage														
between DTP1 & Rota1	19	4.0%	24	4.6%	14	7.0%	13	14.0%	70	6.7%	29	3.7%	10	4.4%
between DTP3 & Rota2-3 (Rotarix & RotaTeq)	19	5.0%	24	6.3%	14	10.8%	13	17.3%	70	8.9%	29	5.5%	10	4.5%
between DTP3 & Rota2 (Rotarix)	14	3.3%	20	2.9%	11	7.2%	5	4.0%	50	4.1%	23	2.9%	10	4.5%
between DTP3 & Rota3 (RotaTeq)	5	9.6%	4	23.5%	3	24.1%	8	25.5%	20	20.9%	6	15.3%	—	—
National Immunization Program Performance for Rotavirus Vaccine Coverage														
Countries with Good Utilization and Good Access (Drop-out < 10% & Rota1 ≥ 80%)	14	74%	16	67%	9	53%	9	64%	48	65%	21	72%	7	70%
Countries with Poor Utilization and Good Access (Drop-out ≥ 10% & Rota1 ≥ 80%)	2	11%	3	13%	3	18%	1	7%	9	12%	3	10%	1	10%
Countries with Good Utilization and Poor Access (Drop-out < 10% & Rota1 < 80%)	0	0%	4	17%	0	0%	1	7%	5	7%	2	7%	2	20%
Countries with Poor Utilization and Poor Access (Drop-out ≥ 10% & Rota1 < 80%)	3	16%	1	4%	2	12%	2	14%	8	11%	3	10%	0	0%

*Note*: Excludes 14 countries (Argentina, Australia, Austria, Ecuador, El Salvador, Iraq, Ireland, Israel, Moldova, Norway, Palau, Paraguay, South Africa, United Kingdom) with missing, incomplete, or inconsistent WHO/UNICEF – Joint Reporting Form (JRF) data for rotavirus vaccine coverages for Rota1 and Rota2-3.

a Vaccine coverage for rotavirus vaccine and DTP reported to the WHO/UNICEF – JRF, 2016.

b Lower Income Countries (LICs) defined as GNI per capita ≤ US$1,005; Lower-middle Income Countries (LMICs) defined as US$1,006 ≤ GNI per capita ≤ US$3,955; Upper-middle Income Countries (UMICs) defined as US$3,956 ≤ GNI per capita ≤ US$12,235; Higher Income Countries (HICs) defined as GNI per capita > US$12,235.

c List of Phase IV countries that receive financial support from Gavi for new vaccine introductions from 2016–2020.

d Includes the five (Bhutan, Honduras, Mongolia, Sri Lanka, Ukraine) Phase III (2011-15) Gavi-graduating countries.

e Country reported Rotavirus vaccine coverage estimates for dose 1 (Rota1) and either dose 2 or 3 (Rota2-3, depending on the schedule) for countries that have reported introducing the rotavirus vaccine into their National Immunization Programs by December 2016, data collected from the WHO/UNICEF – JRF, 2016.

f Drop-out rate between the first and last dose in the vaccine series, calculated using formula: (Coverage first dose – Coverage final dose) / Coverage first dose) × 100.

g DTP coverage for dose 1 (DTP1) and dose3 (DTP3) for countries that have reported introducing the rotavirus vaccine into their National Immunization Program by December 2016, data collected from the WHO/UNICEF – JRF, 2016

h Percent difference between DTP and Rotavirus Vaccines for the first and last dose of the series. Calculated using formula: [(DTP coverage – Rota coverage) / DTP coverage] × 100.

i Performance of delivering the rotavirus vaccine based on four indicators that evaluate service utility and vaccine access based on the calculated rota vaccine drop-out rate and Rota dose 1.

When matching the rotavirus and DTP vaccination schedules for these 70 countries, all countries except for Estonia, Fiji, and Finland had rotavirus vaccine schedules that would align with that of the DTP vaccine. Three countries (São Tomé and Príncipe, Senegal, Zimbabwe) reported a higher Rota1 coverage than DTP1 coverage, while 11 countries (Burundi, Djibouti, Eritrea, Ghana, Guatemala, Panama, Qatar, Sierra Leone, Swaziland, Togo, Zimbabwe) reported a higher last dose coverage for rotavirus than DTP3. Out of this subset of 14 countries, all except for São Tomé and Príncipe were Rotarix-introducing countries of which 7 were LICs, 5 LMICs, 1 UMIC, and 1 HIC. Zimbabwe was the only country to have consistently reported higher rotavirus coverage by 1% compared to DTP1 for the first and DTP3 for the last dose of the series. It would be expected that Rotarix-introducing countries would have a higher Rota2 coverage than DTP3 because of the number of partially immunized infants that tend to drop-out of the vaccine series. Instead, the percent difference between DTP3 and Rota3 were greater among the 20 RotaTeq-introducing countries with complete coverage data (Table 3). Coverage discrepancies between these two vaccines increased from 9.6% to 25.5% when moving across income groups, from LICs to HICs respectively. Within this subset of RotaTeq-introducing countries, 15 had rotavirus vaccine introductions in 2014 or prior, Jordan, Latvia, and Mali introduced rotavirus vaccination in 2015 while Liberia and São Tomé and Príncipe had introductions in 2016. Rotavirus vaccine introduction timelines, therefore, appear not to have accounted for the percent differences between the JRF reported DTP and rotavirus vaccine coverages.

On the other hand, 22 countries (Burkina Faso, Burundi, Djibouti, Eritrea, Ethiopia, Fiji, Gambia, Ghana, Guyana, Honduras, Kiribati, Libya, Morocco, Nicaragua, Panama, Qatar, Rwanda, Sierra Leone, Swaziland, Tanzania, Uzbekistan, Zambia) reported the same DTP1 and Rota1 coverage rates, having zero percent difference between the two vaccines. Similarly, 16 countries (Armenia, Bolivia, Burkina Faso, Fiji, Gambia, Honduras, Libya, Mali, Mauritania, Morocco, Nicaragua, Rwanda, Senegal, Tajikistan, Uzbekistan, Zambia) reported the same Rota2-3 and DTP3 coverages, while 10 countries (Burkina Faso, Fiji, Gambia, Honduras, Libya, Morocco, Nicaragua, Rwanda, Uzbekistan, Zambia) reported the same coverage for rotavirus and DTP vaccines for the first and last dose within the series, averaging 98% and 97% respectively.

The 70 introducing countries that reported rotavirus vaccine coverage had an average Rota1 coverage of 89% (Table 3). Out of all income groups, LICs had the highest rotavirus vaccine coverage for the first and last dose, at 91% and 85% respectively. Following these, LMICs had the second highest coverage rates, at 89% and 83%. UMICs had the lowest rotavirus vaccine coverage for the last dose in the series (80%) and had the highest drop-out rate (11%) out of all income groups (Table 3). Gavi-eligible countries had a slightly higher first dose rotavirus vaccine coverage (91%) compared to Gavi-transitioning countries (90%), but had higher drop-out rates than Gavi-transitioning countries, 8% and 4% respectively (Table 3).

Contrary to DTP vaccine, rotavirus vaccine coverage decreased in higher income groups, as compared to lower income groups. When moving from LICs to HICs, for example, DTP3 vaccine coverage increased from 89% to 97%, respectively, but Rota2-3 vaccine coverage decreased from 85% to 80% (Table 3). Such coverage differences could be a result of an enforced upper age limit on last rotavirus vaccine dose, despite WHO's recommendations to remove the age limit. HICs had the largest discrepancy between reported rotavirus and DTP vaccine coverages and lowest rotavirus vaccine coverage (Table 3).

The ten Eastern Mediterranean reporting countries had the highest Rota2-3 coverage (91%) and the lowest drop-out rates (2%). A great majority of countries in the region (80%, 8/10) also had good access to and utilization of rotavirus vaccine. The African region had the second highest Rota2-3 coverage (84%) but the third highest drop-out rates (8%). Based on the four performance indicators (Appendix 3), a great majority of African region introducing countries (73%, 22/30) had good access to and utilization of rotavirus vaccine. Despite being early introducers, countries in the Americas reported the third highest Rota2-3 coverages (82%) and had the second highest drop-out rate (9%). Only 57% (8/14) of introducing countries in the region with complete coverage data were classified as having good rotavirus vaccine access and utilization. Despite having poor coverage for the last dose in the rotavirus vaccine series (80%), a great majority of introducing European countries (73%, 8/11) had good access to and utilization of the vaccine and the second lowest drop-out rate (6%) out of all WHO regions. In contrast, Western Pacific countries reported the lowest Rota2-3 coverage (62%) with the highest drop-out rate (20%) when compared to other WHO regions. It was also the region with the fewest high-performing countries, where less than half (2/5) presented good access to and utilization of rotavirus vaccine.

LICs had the highest percent (55%) of infants immunized with at least one dose of the rotavirus vaccine and the highest vaccine coverage for the first and last dose, 91% and 85% respectively (Table 3, Table 4). In contrast, HICs had the lowest rotavirus vaccine coverage for the first (84%) and last (80%) dose of rotavirus vaccine, but displayed the lowest drop-out rates (6%). Despite HICs having sub-optimal vaccine coverage values and the highest percent (9%) of unimmunized infants living in introducing countries, 50% of the surviving infant population in HICs lived in introducing countries and received at least one dose of the vaccine, having the second highest percentage of immunized infants following LICs’ 55% of immunized infants (Table 4). Of all income groups, the 36 non-introducing HICs had the lowest percent (41%) of untargeted infants followed by 42% of untargeted infants living in 12 non-introducing LICs (Table 4). Even though almost half (48%) of the global surviving infant population reside in LMICs, only 14% of those infants live in countries that have introduced rotavirus vaccines and received at least one dose of the vaccine (Table 4). LMICs had the highest percent of untargeted infants, with 84% of infants residing in the 25 LMICs that had not introduced rotavirus vaccines. However, among the introducing LMICs, only 2% of infants residing in the 26 introducing countries did not receive any rotavirus vaccine doses.

**Table 4.** Summary of rotavirus immunized, unimmunized, and untargeted infants over the total surviving infant population by WHO region, income-group, and gavi status, 2016.

	Income Groups for 2016 as Defined by the World Bank										GAVI Phase IV			
	LICs (n = 31)		LMICs (n = 51)		UMICs (n = 56)		HICs (n = 56)		Total (n = 194)		Eligible (n = 52)		Transitioning (n = 21)	
Variables	n	%	n	%	n	%	n	%	n	%	n	%	n	%
Sum of Infants Immunized (at least one dose) against Rotavirus (in thousands) residing in Introducing Countries														
Countries	(n = 19)		(n = 26)		(n = 19)		(n = 20)		(n = 84)		(n = 29)		(n = 11)	
Africa	11,850	62%	4,426	35%	1,129	51%	0	0%	17,405	51%	15,174	50%	1,067	83%
Americas	167	68%	1,005	95%	7,267	84%	3,569	75%	12,008	82%	167	68%	532	78%
East Mediterranean	0	0%	2,684	25%	1,148	44%	716	82%	4,548	28%	1,799	21%	0	0%
Europe	0	0%	953	60%	0	0%	1,716	34%	2,669	25%	228	61%	725	53%
Southeast Asia	0	0%	0	0%	0	0%	0	0%	0	0%	0	0%	0	0%
West Pacific	0	0%	4	0.1%	17	0.1%	296	16%	317	1.4%	0	0%	2	0.1%
Total	12,017	55%	9,073	14%	9,560	27%	6,297	50%	36,947	28%	17,369	25%	2,327	23%
Sum of Unimmunized Infants (in thousands) based on Rota1 Coverage Residing in Introducing Countries														
Countries	(n = 19)		(n = 26)		(n = 19)		(n = 20)		(n = 84)		(n = 29)		(n = 11)	
Africa	630	3%	666	5%	114	5%	0	0%	1,410	4%	1,081	4%	214	17%
Americas	79	32%	56	5%	1,128	13%	487	10%	1,749	12%	79	32%	28	4%
East Mediterranean	0	0%	271	3%	143	5%	23	3%	437	3%	257	3%	0	0%
Europe	0	0%	28	2%	0	0%	492	10%	521	5%	7	2%	21	2%
Southeast Asia	0	0%	0	0%	0	0%	0	0%	0	0%	0	0%	0	0%
West Pacific	0	0%	1.4	<0.1%	1.2	<0.1%	72	4%	75	0.3%	0	0%	0.5	<0.1%
Total	709	3%	1,023	2%	1,387	4%	1,075	9%	4,192	3%	1,424	2%	263	3%
Sum of Untargeted Infants (in thousands) that are Residing in Non-Introducing Countries and Presumed Unimmunized														
Countries	(n = 12)		(n = 25)		(n = 37)		(n = 36)		(n = 110)		(n = 23)		(n = 10)	
Africa	6,601	35%	7,520	60%	950	43%	1.4	100%	15,073	44%	14,110	46%	0	0%
Americas	0	0%	0	0%	260	3%	690	15%	950	6%	0	0%	122	18%
East Mediterranean	1,632	100%	7,877	73%	1,325	51%	139	16%	10,973	69%	6,584	76%	0	0%
Europe	0	0%	602	38%	4,249	100%	2,827	56%	7,678	71%	140	37%	620	45%
Southeast Asia	895	100%	32,837	100%	688	100%	0	0%	34,420	100%	28,663	100%	5,068	100%
West Pacific	0	0%	4,629	100%	16,632	100%	1,535	81%	22,796	98%	517	100%	1,787	100%
Total	9,128	42%	53,464	84%	24,105	69%	5,192	41%	91,890	69%	50,014	73%	7,597	75%

*Note:* Rotavirus vaccine coverages imputed for countries with: (1) inconsistent data for El Salvador, Moldova; (2) incomplete or missing data for Australia, Austria, Iraq, Ireland, Israel, Norway, South Africa, United Kingdom.

a Data calculated based on infant mortality, birth rate and total population collected from the United Nations Population Database, 2016.

b Lower Income Countries (LICs) defined as GNI per capita ≤ US$1,005; Lower-middle Income Countries (LMICs) defined as US$1,006 ≤ GNI per capita ≤ US$3,955; Upper-middle Income Countries (UMICs) defined as US$3,956 ≤ GNI per capita ≤ US$12,235; Higher Income Countries (HICs) defined as GNI per capita > US$12,235.

c List of Phase IV countries that receive Gavi financial support for new vaccine introductions from 2016–2020.

d Includes the five (Bhutan, Honduras, Mongolia, Sri Lanka, Ukraine) Phase III (2011-15) Gavi-graduating countries.

e Infants that received at least one dose of the rotavirus vaccine in countries that have introduced the rotavirus vaccine into their National Immunization Program by December 2016.

f Infants that reside in countries that have introduced the rotavirus vaccine before December 2016 but have not been immunized.

g Infants that reside in countries that have not introduced the rotavirus vaccine on or before December 2016.

In the African region, 51% of the region's infant population reside in the 31 introducing countries and have been immunized with at least one dose of rotavirus vaccine; 44% of the region's infants are untargeted because they live in the 16 non-introducing African countries including large countries such as Nigeria and the Democratic Republic of Congo (Table 4). Out of all WHO regions, the Americas had the highest percent (82%) of infants living in introducer countries (18 countries) and immunized with at least one dose of the vaccine, while only 6% of the region's infants reside in the 17 non-introducing countries (Table 4). On the other hand, it was the region with the largest percent (12%) of unimmunized infants within its introducing countries. Only 28% of infants in the Eastern Mediterranean region live in countries that have introduced the vaccine (11 introducing countries) and were immunized with at least one dose of the vaccine while 69% of the infant population resides in the 10 non-introducing countries. Similarly, Europe immunized 25% of its infant population – those residing in the 17 introducing countries – with at least one dose of the vaccine, while 71% of infants in the region reside in the 36 non-introducing countries. In the Western Pacific region, only 1.4% infants live in the 7 introducing countries (5 small island states, Australia, and New Zealand) and have received at least one dose of rotavirus vaccine, while 98% of the region's infant population lives in the remaining 20 non-introducing countries. Around 25% of infants residing in the 29 Gavi-eligible countries have introduced rotavirus vaccines and received at least one rotavirus vaccine dose, while 73% of infants live in the 23 remaining non-introducing Gavi-eligible countries and lack access to the vaccine (Table 4).

### Estimated rotavirus deaths, 2013

Based on the latest 2013 mortality data, there were an estimated 20,718 rotavirus deaths in the 50 countries that had introduced the vaccine by 2013. A further 194,078 deaths took place in the 144 non-introducing countries (Table 5). More than half of the world's infants live in Gavi-eligible countries that have the highest rotavirus mortality rates. A majority (56%) of the estimated rotavirus deaths were in Africa where 93% and 90% of region's deaths were in Gavi-eligible introducing and non-introducing countries, respectively (Table 5). Overall the region had the greatest numbers of deaths (115,021, 54%) with 13,073 (6%) taking place in the 10 introducing countries and an estimated 101,948 (47%) in the 37 non-introducing countries. More than half (54%) the estimated rotavirus deaths occurred in LMICs and 29% in LICs, of which 79% were in Gavi-eligible countries. More than half (54%) of global rotavirus estimated deaths occurred in five countries: India (47,082, 22%), Nigeria (30,800, 14%), Pakistan (14,700, 7%), Democratic Republic of the Congo (13,500, 6%), and Angola (9,682, 5%), with only Angola having introduced the vaccine in 2014.

**Table 5.** Estimated rotavirus deaths in countries with and without rotavirus vaccine introduction on or before 2013 by WHO region and country income-group.

	Income Groups for 2013 as Defined by the World Bank										GAVI Phase IV			
	LICs (n = 33)		LMICs (n = 47)		UMICs (n = 55)		HICs (n = 59)		Total (*N* = 194)		Eligible (n = 52)		Transitioning (n = 21)	
WHO Regions	n	%	n	%	n	%	n	%	n	%	n	%	n	%
Introducing Countries														
Countries	(n = 6)		(n = 14)		(n = 17)		(n = 13)		(n = 50)		(n = 10)		(n = 7)	
Africa	8,588	4%	3,531	2%	954	0.4%	0	0%	13,073	6%	12,119	6%	0	0%
Americas	0	0%	584	0.3%	846	0.4%	163	0.1%	1,593	0.7%	0	0%	312	0.1%
East Mediterranean	0	0%	4,962	2%	1,006	0.5%	64	<0.1%	6,032	3%	4,554	2%	0	0%
Europe	0	0%	4	<0.1%	1	<0.1%	5	<0.1%	10	<0.1%	0	0%	5	<0.1%
Southeast Asia	0	0%	0	0%	0	0%	0	0%	0	0%	0	0%	0	0%
Western Pacific	0	0%	2	<0.1%	7	<0.1%	1	<0.1%	10	<0.1%	0	0%	0	0%
Total	8,588	4%	9,083	4%	2,814	1%	233	0.1%	20,718	10%	16,673	8%	317	0.1%
Non-Introducing Countries														
Countries	(n = 27)		(n = 33)		(n = 38)		(n = 46)		(n = 144)		(n = 42)		(n = 14)	
Africa	49,279	23%	42,257	20%	10,336	5%	76	<0.1%	101,948	47%	91,281	42%	9,829	5%
Americas	800	0.4%	0	0%	12	<0.1%	50	<0.1%	862	0.4%	800	0.4%	2	<0.1%
East Mediterranean	8,303	4%	15,929	7%	301	0.1%	9	<0.1%	24,542	11%	23,003	11%	0	0%
Europe	0	0%	1,184	0.6%	474	0.2%	78	<0.1%	1,736	0.8%	391	0.2%	913	0.4%
Southeast Asia	3,424	1.6%	52,740	25%	124	0.1%	0	0%	56,288	26%	52,247	24%	3,917	2%
Western Pacific	541	0.3%	4,840	2%	3,288	2%	33	<0.1%	8,702	4%	1,351	0.6%	1,485	0.7%
Total	62,347	29%	116,950	54%	14,535	7%	246	0.1%	194,078	90%	169,073	79%	16,146	8%
Global Total	70,935	33%	126,033	59%	17,349	8%	479	0.2%	214,796	100%	185,746	86%	16,463	8%

a Estimated rotavirus caused deaths for children under 5 years, World Health Organization 2013 (17).

b Lower Income Countries (LICs) defined as GNI per capita ≤ US$1,045; Lower-middle Income Countries (LMICs) defined as US$1,046 ≤ GNI per capita ≤ US$4,125; Upper-middle Income Countries (UMICs) defined as US$4,125 ≤ GNI per capita ≤ US$12,745; Higher Income Countries (HICs) defined as GNI per capita > US$12,745.

c List of Phase IV countries that receive Gavi financial support for new vaccine introduction from 2016–2020.

d Includes the five (Bhutan, Honduras, Mongolia, Sri Lanka, Ukraine) Phase III (2011-15) Gavi-graduating countries.

e Countries that introduced the rotavirus vaccine into their National Immunization Program by December 2013, WHO/UNICEF – Joint Reporting Form (JRF), New Vaccine Introduction dataset.

f Countries that have not introduced the rotavirus vaccine into their National Immunization Program by December 2013, WHO/UNICEF – JRF, New Vaccine Introduction dataset.

Following the Africa region, the Southeast Asia region had the second highest estimated rotavirus deaths (56,288, 26%) while Eastern Mediterranean and Western Pacific regions contributed 14% (24,542) and 4% (8,702) of deaths, respectively. In the Eastern Mediterranean region, about half (48%) of the region's estimated rotavirus deaths were in Pakistan. Afghanistan, Somalia, and Sudan, which together accounted for 37% (11,232) of the East Mediterranean region deaths. In the Western Pacific Region, China and the Philippines had the highest estimated rotavirus deaths with 37% (3,191) and 30% (2,599), respectively and account for more than half (66%) of the region's estimated rotavirus deaths. Countries in the Americas (2862, 0.4%) and Europe (1,736, 0.8%) had the lowest numbers of estimated deaths in non-introducing countries. In the Americas, Haiti (800, 33%), Guatemala (249, 10%), Bolivia (193, 8%), and Mexico (192, 8%) accounted for 58% of estimated deaths in the region.

In Europe, approximately 30% (526/1,736) of estimated rotavirus deaths took place in the 14 non-introducing UMICs (Albania, Azerbaijan, Belarus, Bosnia and Herzegovina, Bulgaria, Croatia, Kazakhstan, Macedonia, Montenegro, Romania, Russia, Serbia, Turkey, Turkmenistan). This is the only income-group in the region that has not had a single rotavirus vaccine introduction by 2016. Out of these countries, Azerbaijan was the only country eligible to receive Gavi NVI grants but has not yet introduced rotavirus vaccine. Despite hosting 23% of the regional disease burden, the other 13 non-Gavi countries have also failed to include rotavirus vaccine in their NIPs.

## Discussion

Following WHO's initial 2006 recommendation to introduce the rotavirus vaccine in the Americas and Europe, in socio-economic settings where its effectiveness was tested, 36% (30/84) of introductions took place in the following five years and most of these introductions (50%, 15/30) were in the Americas, where large vaccine clinical trials had taken place in several of the countries in the region. During the next five years, from 2012–2016, 64% (n = 54) of the 84 total introductions occurred, with 13 taking place in Gavi-supported African countries in 2014 alone following WHO's 2013 recommendation to lift the vaccine's age restrictions. It was the largest number of vaccine introductions to have taken place in a year throughout the eleven-year timeline. During this time, the African region had the highest percent (66%, 31/47) of rotavirus vaccine introductions followed by the East Mediterranean region (52%, 11/21) and the Americas (51%, 18/35).

If early introduction had occurred in the five countries with the highest rotavirus mortality (Angola, Democratic Republic of Congo, India, Nigeria, Pakistan), a potential 1.14 million deaths could have been averted from 2006–2013. Angola was the only country out of these top-mortality countries to introduce vaccine before 2016. In March 2016, India launched a phased introduction of its indigenous vaccine ROTAVAC™ (Bharat Biotech, Hyderabad).^19^ Pakistan began a phased rotavirus vaccine introduction in early 2017^20^ and Nigeria's introduction is scheduled for 2018.^21^ The Democratic Republic of Congo has made plans to introduce rotavirus vaccine with Gavi NVI support.^22^

China has approximately 69% of the Western Pacific infant population (12% of the global cohort). Although it has had a nationally licensed monovalent vaccine (Lanzhou Institute of Biological products), this vaccine has not been introduced into China's NIP. Lanzhou Institute is in late stage development of a new trivalent vaccine and the Wuhan Institute of Biological Products, in collaboration with PATH, is developing a hexavalent vaccine.^19^

Despite having the second highest regional estimates of rotavirus deaths, no country in the Southeast Asia WHO region had introduced the vaccine universally during 2006–2016. In the region, the estimated rotavirus deaths in India and Indonesia alone make up 24% of the 2013 estimated global death toll. As noted above India started a phased introduction of its ROTAVAC™ vaccine in 2016, and will also use its second indigenous vaccine (ROTASIIL™, Serum Institute of India, Pune) in this national rollout planned in 2018. Bharat Biotech received WHO pre-qualification for ROTAVAC™ in January 2018.^2^ Serum Institute of India is also seeking WHO pre-qualification for ROTASIIL™. Indonesia's national vaccine manufacturer, PT Biofarma, Bandung, is developing a rotavirus vaccine in collaboration with the Murdoch Children's Research Institute, Melbourne^19^ but whether Indonesia would consider other WHO pre-qualified rotavirus vaccines in the interim for universal use is uncertain. None of the 13 Gavi-eligible countries in WHO's Southeast Asia and Western Pacific regions had introduced rotavirus vaccine by December 2016. This is in contrast to the 69% of Gavi-eligible African countries to do so. If this hesitation to introduce the existing vaccines was based on vaccine price and the estimated cost-effectiveness of the two multinational vaccines, then the lower-priced Indian vaccines might become attractive alternatives for the region.

Coverage and drop-out rate data show that introducing African countries provide good ‘access’ to the rotavirus vaccine but there is poorer ‘utilization’, as reflected in the higher drop-out rates, i.e. infants start but do not complete the full rotavirus vaccine course. However, these metrics of access and utilization are used to reflect performance of immunization services in general, rather than reflecting use of individual vaccines. Our results show differences between these metrics when they are used to compare rotavirus and DTP vaccines. The implication of this are uncertain but if rotavirus vaccine appears to ‘underperform’ compared to DTP vaccines this could indicate a need to strengthen health systems in relation to a particular vaccine program, and may indicate stock-outs or other factors affecting the vaccine's integration into the NIP. Further analysis of discrepancies between these metrics for different vaccines with the same schedule could be of interest, including the analysis of other factors within the NIP and vaccine delivery system that could impact the access and utilization of vaccines. Gavi financially supports eligible and transitioning countries with health systems strengthening grants (HSS). According to Gavi's annual reported commitments and disbursements, only 33% (13/39) of its rotavirus introducing countries received HSS grants, including 31% (9/29, Djibouti, Eritrea, Haiti, Guinea-Bissau, Mauritania, Rwanda, Sudan, Tajikistan, Yemen) and 40% (4/10, Angola, Bolivia, Congo, Nicaragua) of rotavirus introducing Gavi-eligible and Gavi-transitioning countries, respectively, received such financial support.^22^

This review suggests that HICs should increase rotavirus vaccine coverage. The sub-optimal Rota1 coverage could be a reflection of low perceived risk of disease. A recent review has shown the positive impact of rotavirus vaccines on morbidity in countries of all income groups, including HICs.^23^ Safety concerns around intussusception could also be a contributing factor affecting the uptake of rotavirus vaccine into NIPs of HIC countries. Actual and perceived cost of vaccine in non-Gavi eligible and transitioning countries may explain why HICs and UMICs have been slower to introduce rotavirus vaccines.^24^ UMICs had the lowest overall percent (34%) of rotavirus vaccine introductions. About 33% of countries in the Western Pacific region are UMICs and only two non-Gavi countries have introduced the vaccine. Excluding Gavi's financial support efforts in the Africa region, the UMICs in the Americas had the second highest percent (55%) of UMICs rotavirus vaccine introducers and the largest number of UMICs introducers overall (61%, 11/18). More than half (57%) of the Americas is made up of UMICs. These vaccine introductions could be attributable to the Pan-American Health Organization's (PAHO) Revolving Fund, a regional pooled procurement mechanism with a fixed vaccine pricing.^24^^,^^25^ This means that countries, irrespective of their GNI per capita, have access to the same vaccines and at the same affordable price. Gulf cooperation countries (GCC) in the EMRO region have also established a limited pooled procurement mechanism.^25^ Unfortunately, UMICs in other regions do not have access to such pooled procurement mechanisms and typically need to negotiate vaccine prices individually with manufacturers.

This review has a number of limitations. It utilized secondary data sources which had some inconsistencies between the different datasets. For example, not all countries that introduced rotavirus vaccine reported vaccine coverage values for 2016. Data were imputed for those flagged observations, potentially underestimating or overestimating the percent of infants that were immunized against rotavirus. Vaccine coverage data from the private sector were also not considered, likely underestimating the number of infants immunized in introducing and non-introducing countries. In addition, estimated rotavirus deaths were only available for 2013. In an attempt to reflect the decreased disease burden after rotavirus vaccine introductions, the number of estimated rotavirus deaths were disaggregated by whether countries had introduced the vaccine by 2013 or not. Absence of coverage data collected for DTP2 also limited any further comparison between the coverage percent difference of rotavirus and DTP vaccines. WUENIC estimates for the first dose of the rotavirus vaccine were also not available, limiting the comparison between country-reported and WUENIC vaccine coverage estimates. This could impact on the reliability of our estimates of ‘access and utilization’ of the rotavirus vaccine i.e. WUENIC estimates differ from country-reported estimates. Other potential data quality issues of JRF-reported rotavirus and DTP vaccine coverages also impose a limitation on the study's results and are difficult to quantify. For example, countries with poor and outdated census data may either inflate or underestimate the vaccine coverage denominator. Poor use and recording of the immunization card affects a country's ability to quantify the number of infants immunized, the numerator of the vaccine coverage. Such data quality issues would affect estimates of coverage and the calculated percent difference between the DTP and rotavirus vaccines.

In summary, a great deal of progress was made in increasing access to rotavirus vaccine globally. Nevertheless by December 2016, despite WHO's 2009 recommendation, the vaccine had not been introduced into the NIPs of 110 countries, representing 69% of the world's children. To prevent the continued mortality, morbidity, and the enormous economic burden caused by rotavirus disease, efforts should be made to increase advocacy and communicate the importance of this disease and establish alternate financing mechanisms for countries that are not Gavi eligible or have transitioned from Gavi. The over-representation of African countries within the Gavi subset, and the fact that most countries in the region have high estimated rotavirus deaths, likely explains why efforts have been geared towards introducing the rotavirus vaccine in this region. If the global rotavirus death toll is to be further decreased, future efforts should be focused on expediting rotavirus vaccine introduction in the Southeast Asia WHO region although important progress has been made in India. In addition, focused efforts should be made to increase the coverage, utilization, and access of the vaccine in countries that have already introduced it. If we can successfully make these efforts moving forward, we will see important reductions in the burden of rotavirus worldwide. This review emphasizes how Gavi has played a major role in helping low-income countries (LICs and LMICs) improve access to new and under-utilized vaccines, saving millions of lives from vaccine preventable diseases. Yet there is still a need to further increase vaccine access and coverage to rotavirus vaccine, in Gavi and non-Gavi countries alike. On average HICs had poorer rotavirus vaccine coverage in comparison to LICs and (LMICs) that usually have weaker health infrastructures when compared to richer countries. We should therefore continue the momentum with Gavi-eligible countries, and make strident efforts to provide countries of all income groups with access to affordable vaccines through alternate financing mechanisms.

## Figures and Tables

**Table A1.** Classification of 194 countries according to WHO Region, income group, Gavi-eligibility status and rotavirus vaccine introduction status as of December 2016.

WHO Regions	Country	Income group^b^	Gavi Phase IV status^c^	Rotavirus vaccine introduction status^d^
AFRO	Benin^d^	LIC	Eligible	Non-Introducer
	Burkina Faso	LIC	Eligible	Introducer
	Burundi	LIC	Eligible	Introducer
	Central African Republic^d^	LIC	Eligible	Non-Introducer
	Chad	LIC	Eligible	Non-Introducer
	Comoros	LIC	Eligible	Non-Introducer
	Democratic Republic of Congo	LIC	Eligible	Non-Introducer
	Eritrea	LIC	Eligible	Introducer
	Ethiopia	LIC	Eligible	Introducer
	Gambia	LIC	Eligible	Introducer
	Guinea^d^	LIC	Eligible	Non-Introducer
	Guinea Bissau	LIC	Eligible	Introducer
	Liberia	LIC	Eligible	Introducer
	Madagascar	LIC	Eligible	Introducer
	Malawi	LIC	Eligible	Introducer
	Mali	LIC	Eligible	Introducer
	Mozambique^e^	LIC	Eligible	Introducer
	Niger	LIC	Eligible	Introducer
	Rwanda	LIC	Eligible	Introducer
	Senegal	LIC	Eligible	Introducer
	Sierra Leone	LIC	Eligible	Introducer
	South Sudan	LIC	Eligible	Non-Introducer
	Tanzania	LIC	Eligible	Introducer
	Togo	LIC	Eligible	Introducer
	Uganda^d^	LIC	Eligible	Non-Introducer
	Zimbabwe	LIC	Eligible	Introducer
	Angola	LMIC	Transitioning	Introducer
	Cabo Verde	LMIC	—	Non-Introducer
	Cameroon	LMIC	Eligible	Introducer
	Congo	LMIC	Transitioning	Introducer
	Côte d'Ivoire^d^	LMIC	Eligible	Non-Introducer
	Ghana	LMIC	Eligible	Introducer
	Kenya	LMIC	Eligible	Introducer
	Lesotho^d^	LMIC	Eligible	Non-Introducer
	Mauritania	LMIC	Eligible	Introducer
	Nigeria^d^	LMIC	Eligible	Non-Introducer
	São Tomé and Príncipe	LMIC	Eligible	Introducer
	Swaziland	LMIC	—	Introducer
	Zambia	LMIC	Eligible	Introducer
	Algeria	UMIC	—	Non-Introducer
	Botswana	UMIC	—	Introducer
	Equatorial Guinea	UMIC	—	Non-Introducer
	Gabon	UMIC	—	Non-Introducer
	Mauritius	UMIC	—	Introducer
	Namibia	UMIC	—	Introducer
	South Africa	UMIC	—	Introducer
	Seychelles^d^	HIC	—	Non-Introducer
AMRO	Haiti	LIC	Eligible	Introducer
	Bolivia	LMIC	Transitioning	Introducer
	El Salvador	LMIC	—	Introducer
	Guatemala	LMIC	—	Introducer
	Honduras	LMIC	Graduating	Introducer
	Nicaragua	LMIC	Transitioning	Introducer
	Argentina	UMIC	—	Introducer
	Belize^d^	UMIC	—	Non-Introducer
	Brazil	UMIC	—	Introducer
	Colombia	UMIC	—	Introducer
	Costa Rica	UMIC	—	Non-Introducer
	Cuba	UMIC	Transitioning	Non-Introducer
	Dominica	UMIC	—	Non-Introducer
	Dominican Republic	UMIC	—	Introducer
	Ecuador	UMIC	—	Introducer
	Grenada	UMIC	—	Non-Introducer
	Guyana	UMIC	Transitioning	Introducer
	Jamaica	UMIC	—	Non-Introducer
	Mexico	UMIC	—	Introducer
	Panama	UMIC	—	Introducer
	Paraguay	UMIC	—	Introducer
	Peru	UMIC	—	Introducer
	Saint Lucia	UMIC	—	Non-Introducer
	Saint Vincent and the Grenadines	UMIC	—	Non-Introducer
	Suriname	UMIC	—	Non-Introducer
	Venezuela	UMIC	—	Introducer
	Antigua and Barbuda	HIC	—	Non-Introducer
	Bahamas^d^	HIC	—	Non-Introducer
	Barbados	HIC	—	Non-Introducer
	Canada^d^	HIC	—	Non-Introducer
	Chile	HIC	—	Non-Introducer
	Saint Kitts and Nevis	HIC	—	Non-Introducer
	Trinidad and Tobago	HIC	—	Non-Introducer
	United States	HIC	—	Introducer
	Uruguay	HIC	—	Non-Introducer
EMRO	Afghanistan^d^	LIC	Eligible	Non-Introducer
	Somalia	LIC	Eligible	Non-Introducer
	Djibouti	LMIC	Eligible	Introducer
	Egypt	LMIC	—	Non-Introducer
	Jordan	LMIC	—	Introducer
	Morocco	LMIC	—	Introducer
	Pakistan^d^	LMIC	Eligible	Non-Introducer
	Sudan	LMIC	Eligible	Introducer
	Syria	LMIC	—	Non-Introducer
	Tunisia	LMIC	—	Non-Introducer
	Yemen	LMIC	Eligible	Introducer
	Iran	UMIC	—	Non-Introducer
	Iraq	UMIC	—	Introducer
	Lebanon	UMIC	—	Non-Introducer
	Libya	UMIC	—	Introducer
	Bahrain	HIC	—	Introducer
	Kuwait	HIC	—	Non-Introducer
	Oman	HIC	—	Non-Introducer
	Qatar	HIC	—	Introducer
	Saudi Arabia	HIC	—	Introducer
	United Arab Emirates	HIC	—	Introducer
EURO	Armenia	LMIC	Transitioning	Introducer
	Georgia	LMIC	Transitioning	Introducer
	Kyrgyzstan	LMIC	Eligible	Non-Introducer
	Moldova	LMIC	Transitioning	Introducer
	Tajikistan	LMIC	Eligible	Introducer
	Ukraine	LMIC	Graduating	Non-Introducer
	Uzbekistan	LMIC	Transitioning	Introducer
	Albania	UMIC	—	Non-Introducer
	Azerbaijan	UMIC	Transitioning	Non-Introducer
	Belarus	UMIC	—	Non-Introducer
	Bosnia Herzegovina	UMIC	—	Non-Introducer
	Bulgaria	UMIC	—	Non-Introducer
	Croatia	UMIC	—	Non-Introducer
	Kazakhstan	UMIC	—	Non-Introducer
	Macedonia	UMIC	—	Non-Introducer
	Montenegro	UMIC	—	Non-Introducer
	Romania^d^	UMIC	—	Non-Introducer
	Russia^d^	UMIC	—	Non-Introducer
	Serbia	UMIC	—	Non-Introducer
	Turkey	UMIC	—	Non-Introducer
	Turkmenistan	UMIC	—	Non-Introducer
	Andorra	HIC	—	Non-Introducer
	Austria	HIC	—	Introducer
	Belgium	HIC	—	Introducer
	Cyprus	HIC	—	Non-Introducer
	Czech Republic	HIC	—	Non-Introducer
	Denmark	HIC	—	Non-Introducer
	Estonia	HIC	—	Introducer
	Finland	HIC	—	Introducer
	France	HIC	—	Non-Introducer
	Germany	HIC	—	Introducer
	Greece	HIC	—	Introducer
	Hungary	HIC	—	Non-Introducer
	Iceland	HIC	—	Non-Introducer
	Ireland^e^	HIC	—	Introducer
	Israel	HIC	—	Introducer
	Italy^d^	HIC	—	Non-Introducer
	Latvia	HIC	—	Introducer
	Lithuania	HIC	—	Non-Introducer
	Luxembourg	HIC	—	Introducer
	Malta	HIC	—	Non-Introducer
	Monaco	HIC	—	Non-Introducer
	Netherlands	HIC	—	Non-Introducer
	Norway^e^	HIC	—	Introducer
	Poland^d^	HIC	—	Non-Introducer
	Portugal	HIC	—	Non-Introducer
	San Marino	HIC	—	Non-Introducer
	Slovakia	HIC	—	Non-Introducer
	Slovenia	HIC	—	Non-Introducer
	Spain	HIC	—	Non-Introducer
	Sweden^d^	HIC	—	Non-Introducer
	Switzerland	HIC	—	Non-Introducer
	United Kingdom	HIC	—	Introducer
SEARO	DPR Korea	LIC	Eligible	Non-Introducer
	Nepal	LIC	Eligible	Non-Introducer
	Bangladesh	LMIC	Eligible	Non-Introducer
	Bhutan	LMIC	Graduating	Non-Introducer
	India^d^	LMIC	Eligible	Non-Introducer
	Indonesia	LMIC	Transitioning	Non-Introducer
	Myanmar	LMIC	Eligible	Non-Introducer
	Sri Lanka	LMIC	Graduating	Non-Introducer
	Timor-Leste	LMIC	Transitioning	Non-Introducer
	Maldives	UMIC	—	Non-Introducer
	Thailand^d^	UMIC	—	Non-Introducer
WPRO	Cambodia	LMIC	Eligible	Non-Introducer
	Kiribati	LMIC	Transitioning	Introducer
	Lao PDR	LMIC	Eligible	Non-Introducer
	Micronesia	LMIC	—	Introducer
	Mongolia	LMIC	Graduating	Non-Introducer
	Papua New Guinea	LMIC	Transitioning	Non-Introducer
	Philippines^d^	LMIC	—	Non-Introducer
	Solomon Islands	LMIC	Eligible	Non-Introducer
	Vanuatu	LMIC	—	Non-Introducer
	Vietnam	LMIC	Transitioning	Non-Introducer
	China	UMIC	—	Non-Introducer
	Fiji	UMIC	—	Introducer
	Malaysia	UMIC	—	Non-Introducer
	Marshall Islands	UMIC	—	Introducer
	Nauru	UMIC	—	Non-Introducer
	Niue	UMIC	—	Non-Introducer
	Samoa	UMIC	—	Non-Introducer
	Tonga	UMIC	—	Non-Introducer
	Tuvalu	UMIC	—	Non-Introducer
	Australia	HIC	—	Introducer
	Brunei Darussalam	HIC	—	Non-Introducer
	Cook Islands	HIC	—	Non-Introducer
	Japan	HIC	—	Non-Introducer
	Korea	HIC	—	Non-Introducer
	New Zealand	HIC	—	Introducer
	Palau	HIC	—	Introducer
	Singapore^d^	HIC	—	Non-Introducer

^a^Lower Income Countries (LICs) defined as GNI per capita ≤ US$1,045; Lower-middle Income Countries (LMICs) defined as US$1,046 ≤ GNI per capita ≤ US$4,125; Upper-middle Income Countries (UMICs) defined as US$4,125 ≤ GNI per capita ≤ US$12,745; Higher Income Countries (HICs) defined as GNI per capita > US$12,745.^10^^,^^11^

^b^List of Phase IV countries that receive Gavi financial support for new vaccine introduction from 2016-2020 and five Phase III (2011-15) Gavi countries that graduated in Phase IV. Information on website accessed on August 2017.^8^^,^^9^

^c^Rotavirus vaccine introduction status was defined by the reported year of rotavirus vaccine introduction collected from the WHO/UNICEF - Joint Reporting Form (JRF) "Year of New Vaccine Introduction" dataset downloaded on August 2017.^16^

^d^Countries excluded from the final list of introducing countries because although they reported having inclusion of rotavirus vaccine in the National Immunization Schedule or reported coverage of rotavirus vaccine to the WHO/UNICEF - JRF datasets, they did not have a year of rotavirus vaccine introduction in the WHO/UNICEF - JRF, "Year of New Vaccine Introduction" dataset.

^e^Countries included in the final list of introducing countries because they reported the year of rotavirus vaccine introduction but had incomplete WHO/UNICEF - JRF data for either "Rotavirus Vaccine Coverage" or "National Immunization Schedule" datasets downloaded on August 2017.^16^

**Table A2.** Comparing WHO/UNICEF - Joint Reporting Form (JRF) Datasets

WHO/UNICEF - JRF Datasets	Data Collected	Reporting Countries	Countries with Complete^d^ Data	Countries with Incomplete^e^ Data	List of Countries with Incomplete Data
Vaccine Schedule^a^	Rotavirus vaccine included in National Immunization Programs, 2016	100	81	19	Afghanistan, Bahamas, Belize, Benin, Canada, Central African Republic, Cote d'Ivoire, Guinea, India, Italy, Lesotho, Nigeria, Norway*, Romania, Russia, Seychelles, Sweden, Thailand, Uganda
Country Reported Vaccine Coverage Estimates^b^	Rotavirus vaccine coverage for Rota1 and Rota2/3 reported at least once from 2008-2016	89	81	8	Bahamas, India, Italy, Mozambique*, Pakistan, Philippines, Poland, Singapore
New Vaccine Introduction Year^c^	Year of nation-wide rotavirus vaccine introduction reported	84	81	3	Ireland^*^, Mozambique^*^, Norway^*^

^*^Countries defined as rotavirus vaccine introducers (N=84) for reporting year of rotavirus vaccine introduction despite having incomplete data for the other two WHO/UNICEF - JRF datasets

^a^Inclusion of rotavirus vaccine in National Immunization Programs collected from the WHO/UNICEF - JRF dataset, downloaded on July 7, 2017

^b^Country reported rotavirus vaccine coverage estimates for Rota1 and Rota2-3 reported from 2008-16 collected from the WHO/UNICEF - JRF dataset, downloaded on July 5, 2017

^c^Year of rotavirus vaccine introduction dataset collected from the WHO/UNICEF - JRF Year Vaccine Introduction dataset, downloaded on July 6, 2017

^d^Countries having complete data for all three WHO/UNICEF - JRF datasets

^e^Countries with missing data for at least one of the WHO/UNICEF - JRF datasets

**Table A3.** Immunization Service Indicators on Performance.^1^

	Performance Status	
Service Indicators	Good	Poor
**Access:** Defined by Vaccine Coverage of Dose 1	Coverage ≥ 80%	Coverage < 80%
**Utilization:** Defined by Drop-out between Dose 1 & 3	Drop-out < 10%	Drop-out ≥ 10%

^1^As defined by the World Health Organization's immunization performance indicators on access and utilization, conventionally using the coverage and drop-out rates of the diphtheria, tetanus, pertussis (DTP) vaccine as a benchmark.^17^

## Key findings

• Proportionately more rotavirus vaccine introductions have taken place in low- and lower-middle- income countries in the World Health Organization African region with Gavi financial support

• Rotavirus vaccine introductions in low-income countries and lower-middle-income countries had higher coverage rates than introducing countries in other income groups

• Rotavirus vaccine introductions have been slow in the Southeast Asian World Health Organization region despite the proportionately high estimated number of rotavirus deaths

• Rotavirus vaccine introductions have also been slow in the World Health Organization Europe region and upper-middle income countries that are not eligible to receive Gavi new vaccine introduction grants

• Across all income groups, the vaccine coverage for the first and last dose of the rotavirus vaccine series was lower than that of the Diphtheria Tetanus Pertussis vaccine while rotavirus drop-out rates were overall higher than Diphtheria Tetanus Pertussis drop-out rates
